# The complete chloroplast genome sequence of *Trapa kozhevnikoviorum* Pshenn. (Lythraceae)

**DOI:** 10.1080/23802359.2021.1927873

**Published:** 2021-05-19

**Authors:** Godfrey Kinyori Wagutu, Xiangrong Fan, Wuchao Wang, Wei Li, Yuanyuan Chen

**Affiliations:** a Key Laboratory of Aquatic Botany and Watershed Ecology, Wuhan Botanical Garden, Chinese Academy of Sciences, Wuhan, China; b Wuhan Botanical Garden, University of Chinese Academy of Sciences, Beijing, China; c Sino-Africa Joint Research Center, Chinese Academy of Sciences, Wuhan, China; d College of Science, Tibet University, Lhasa, Tibet Autonomous Region, China; e Research Center for Ecology and Environment of Qinghai-Tibetan Plateau, Tibet University, Lhasa, Tibet Autonomous Region, China; f Key Laboratory of Aquatic Botany and Watershed Ecology, Wuhan Botanical Garden, Center of Plant Ecology, Core Botanical Gardens, Chinese Academy of Sciences, Wuhan, China

**Keywords:** *Trapa kozhevnikoviorum*, chloroplast, phylogenetic, Lythraceae

## Abstract

*Trapa* (Lythraceae) is an economically important aquatic genus used for food and medicine, with wide distribution in Asia, Africa, and Europe. Identification of species, genetic studies and utilization of *Trapa* are limited by lack of molecular data. Herein, we report the complete chloroplast (cp) genome sequence of a wild species, *Trapa kozhevnikoviorum* Pshenn. The cp genome size of *T. kozhevnikoviorum* is 155,545 bp, consisting of a pair of inverted repeat regions (IRa/IRb) of length 24,388 bp, separated by the small single copy (SSC) region of 18,275 bp and a large single copy (LSC) region of 88,494 bp. A total of 113 unique genes, including 79 protein-coding, 30 tRNA, and four rRNA were annotated. Phylogenetic analysis based on 15 whole cp genomes of Lythraceae species supported the monophyletic clustering of *Trapa*. A cladal relationship among *T. kozhevnikoviorum*, *T. bicornis*, and *T. natans* was revealed.

## 1. Background

*Trapa* L., commonly known as water chestnut, is a genus in the family Lythraceae, including approximately 30 species distributed across the temperate and subtropical regions of Asia, Africa, and Europe (Chen et al. 2007). *Trapa* is an economically important genus used for food in China and India because of the high protein and starch content in seeds. Its seed coat is used as an antimicrobial medical resource in many other countries (Karg 2006; Artyukhin et al. 2019). The complexity of morphological characteristics of *Trapa* species has created a taxonomical challenge among researchers world-wide, although previous studies agreed that fruit size was a crucial classification criterion (Takano and Kadono 2005; Fan et al. 2016, 2021). Wetland degradation due to human activity and climate change has led to population size decrease in some *Trapa* taxa, and a few of them are endangered (Batsatsashvili and Machutadze 2014; Frey et al. 2017). Among such *Trapa* taxa, *T. kozhevnikoviorum* is a four-horned water chestnut with large fruit, which is sporadically distributed in Tumen River Basin, the border between Russia and China. Only four natural populations of *T. kozhevnikoviorum* were found in recent field investigations (Xue et al. 2016), which makes the conservation of extant populations and their genetic information a concern. Here, we report the complete chloroplast (cp) genome sequence of *T. kozhevnikoviorum* and its phylogenetic position among other species within Lythraceae.

## 2. Methods

Samples of *T. kozhevnikoviorum* were collected from Jixi City, Heilongjiang Province, China (N46°53′9.5″, E133°3′8.9″). The voucher specimen has been deposited in the Herbarium of Wuhan Botanical Garden (voucher number: yychen20180042; Yuanyuan Chen, yychen@wbgcas.cn). DNA was extracted from 0.3 g silica-dried leaf tissue using the CTAB protocol with minor modification of 3 × CTAB buffer used (Doyle and Doyle 1987). DNA library construction and sequencing were performed at the Novogene Co. Ltd. (Beijing, China). DNA libraries were prepared with an insert size of 350 bp using NEBNext Ultra DNA Library Prep Kit. Paired-end sequencing (150 bp reads) was performed on an Illumina NovaSeq 6000 platform (San Diego, CA). The cp genome was assembled using GetOrganelle v1.7.1 with default parameters (Jin et al. 2020). The resultant genome was annotated using plastid genome annotator (PGA) (Qu et al. 2019) with *T. maximowiczii* (NC037023) and *T. bicornis* (NC049010) as reference genomes. Geneious 2020.2.3 (www.geneious.com) was used for further manual annotation with reference to *T. bicornis*. The annotated cp genome was deposited in GenBank with accession number MW027640. Using DnaSP v.6 (Rozas et al. 2017) and a python script (https://www.biostars.org/p/119214/), we identified the insertions/deletions (indels) and single nucleotide polymorphisms (SNPs) between *T. kozhevnikoviorum* and the three published *Trapa* species.

The phylogenetic relationship was constructed using maximum-likelihood (ML) method based on 15 whole cp genomes within the family Lythraceae, with *Ludwigia octovalvis* (NC031385) as an outgroup. The 16 cp genomes were aligned using MAFFT v. 7.471 (Katoh and Standley 2013). The optimal model, TVM + I+G, was obtained from jModelTest2 (Darriba et al. 2012). The ML analysis was performed using PhyML v.3.0 (Guindon et al. 2010) with 1000 bootstrap iterations.

## 3. Results

The cp genome of *T. kozhevnikoviorum* exhibited a typical quadripartite structure of length 155,545 bp, consisting of a pair of inverted repeat regions (IRa/IRb) of length 24,388 bp each, separated by a small single copy (SSC) region of 18,275 bp and a large single copy (LSC) region of 88,494 bp. The GC contents of IR regions, SSC, and LSC were 42.8%, 30.2%, and 34.2%, respectively, with a total GC content of 36.4%. The cp genome encoded a total of 130 genes, including 113 unique genes (79 protein-coding, 30 tRNA, and four rRNA) and 17 duplicated genes (six protein-coding genes (PCGs), seven tRNA genes, and four rRNA genes). Eleven PCGs contained one intron, and two PCGs (*ycf3* and *clp*P) contained two introns. There were minor differences among large-fruit species, with 27 indels and 60 SNPs between *T. kozhevnikoviorum* and *T. natans*, and 23 indels and 66 SNPs between *T. kozhevnikoviorum* and *T. bicornis*. Conversely, obvious differences, with 236 indels and 1412 SNPs, were found between *T. kozhevnikoviorum* and the small-fruit *T. maximowiczii*.

**Figure 1.**
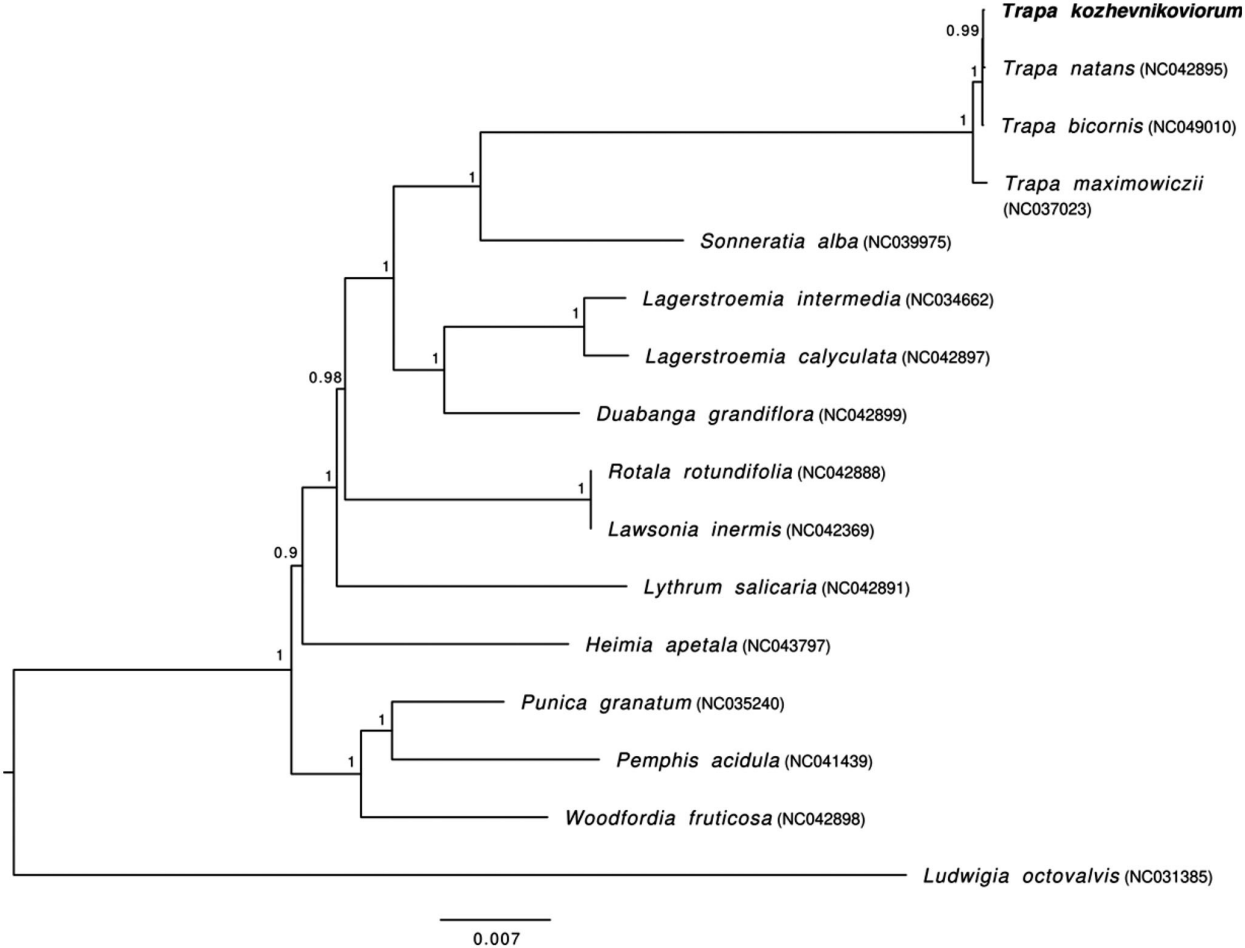
Phylogenetic tree using maximum-likelihood (ML) based on 15 whole chloroplast genomes of Lythraceae species with *Ludwigia octovalvis* as an outgroup. Numbers near the nodes represent ML bootstrap values.

The phylogenetic tree supported the close relationship between *Trapa* and *Sonneratia*, which was reported in previous studies (Yu et al. 2018; Gu et al. 2019; Sun et al. 2020). Monophyletic clustering of *Trapa* was revealed, with the large-fruit *Trapa* species (*T. kozhevnikoviorum*, *T. bicornis*, and *T. natans*) forming a clade closely related to *T. maximowiczii* with small fruit (Figure 1), suggesting the distinct genetic divergence between the two clades, and the basal classification status of the small-fruit species *T. maximowiczii*.

## Data Availability

All datasets for this study are openly available in GenBank with accession code MW027640, https://www.ncbi.nlm.nih.gov/nuccore/MW027640.1/.

## References

[CIT0001] Artyukhin AE, Mikhaylova EV, Kuluev BR. 2019. Genetic variation of water caltrop (*Trapa* L.) in several Russian populations. Plant Genetic Resour Breed Produc Functional Nutraceutical Food, 44–45.

[CIT0002] Batsatsashvili K, Machutadze I. 2014. *Trapa colchica*. The IUCN red list of threatened species, e.T200581A2670883.

[CIT0003] Chen JR, Ding BY, Funston AM. 2007. Flora of China. Vol. 13. Beijing: Science Press, St. Louis: Missouri Botanical Garden Press.

[CIT0004] Darriba D, Taboada GL, Doallo R, Posada D. 2012. jModelTest 2: more models, new heuristics and parallel computing. Nat Methods. 9(8):772. 10.1038/nmeth.2109 PMC4594756 22847109

[CIT0005] Doyle JJ, Doyle JL. 1987. A rapid DNA isolation procedure for small quantities of fresh leaf tissue. Phytochem Bull. 19:11–15.

[CIT0006] Fan XR, Li Z, Chu HJ, Li W, Liu YL, Chen YY. 2016. Analysis of morphological plasticity of *Trapa* L. from China and their taxonomic significance. Plant Sci J. 34(3):340–351.

[CIT0007] Fan XR, Wang WC, Chen L, Li W, Chen YY. 2021. Genetic relationship among 12 *Trapa* species/varietas from Yangtze River Basin revealed by AFLP markers. Aquat Bot. 168:103320.

[CIT0008] Frey D, Reisch C, Narduzzi-Wicht B, Baur EM, Cornejo C, Alessi M, Schoenenberger N. 2017. Historical museum specimens reveal the loss of genetic and morphological diversity due to local extinctions in the endangered water chestnut *Trapa natans* L. (Lythraceae) from the southern Alpine lake area. Bot J Linn Soc. 185(3):343–358.

[CIT0009] Gu C, Ma L, Wu Z, Chen K, Wang Y. 2019. Comparative analyses of chloroplast genomes from 22 Lythraceae species: inferences for phylogenetic relationships and genome evolution within Myrtales. BMC Plant Biol. 19(1):281. 31242865 10.1186/s12870-019-1870-3 PMC6595698

[CIT0010] Guindon S, Dufayard JF, Lefort V, Anisimova M, Hordijk W, Gascuel O. 2010. New algorithms and methods to estimate maximum-likelihood phylogenies: assessing the performance of PhyML 3.0. Syst Biol. 59(3):307–321. 20525638 10.1093/sysbio/syq010

[CIT0011] Jin JJ, Yu WB, Yang JB, Song Y, dePamphilis CW, Yi TS, Li DZ. 2020. GetOrganelle: a fast and versatile toolkit for accurate de novo assembly of organelle genomes. Genome Biol. 21(1):241. 32912315 10.1186/s13059-020-02154-5 PMC7488116

[CIT0012] Karg S. 2006. The water chestnut (*Trapa natans* L.) as a food resource during the 4th to 1st millennia BC at Lake Federsee, Bad Buchau (southern Germany). Environ Archaeol. 11(1):125–130.

[CIT0013] Katoh K, Standley DM. 2013. MAFFT multiple sequence alignment software version 7: improvements in performance and usability. Mol Biol Evol. 30(4):772–780. 23329690 10.1093/molbev/mst010 PMC3603318

[CIT0014] Qu XJ, Moore MJ, Li DZ, Yi TS. 2019. PGA: a software package for rapid, accurate, and flexible batch annotation of plastomes. Plant Methods. 15(1):50. 31139240 10.1186/s13007-019-0435-7 PMC6528300

[CIT0015] Rozas J, Ferrer-Mata A, Sánchez-DelBarrio JC, Guirao-Rico S, Librado P, Ramos-Onsins SE, Sánchez-Gracia A. 2017. DnaSP 6: DNA sequence polymorphism analysis of large data sets. Mol Biol Evol. 34(12):3299–3302. 29029172 10.1093/molbev/msx248

[CIT0016] Sun F, Yin Y, Xue B, Zhou R, Xu J. 2020. The complete chloroplast genome sequence of *Trapa bicornis* Osbeck (Lythraceae). Mitochondrial DNA Part B. 5(3):2746–2747. 33457932 10.1080/23802359.2020.1788432 PMC7782891

[CIT0017] Takano A, Kadono Y. 2005. Allozyme variations and classification of *Trapa* (Trapaceae) in Japan. Aquat Bot. 83(2):108–118.

[CIT0018] Xue J, Xue Z, Wang R, Rubtsova TA, Pshennikova LM, Guo Y. 2016. Distribution pattern and morphological diversity of *Trapa* L. in the Heilong and Tumen River Basin. Plant Sci J. 34(4):506–520.

[CIT0019] Yu T, Hinsinger DD, Strijk JS, Wee AKS. 2018. The first complete chloroplast genome of a major mangrove species *Sonneratia alba* Sm. and its implications on conservation efforts. Mitochondrial DNA Part B. 3(2):500–502. 33474220 10.1080/23802359.2018.1463828 PMC7799946

